# Confidence ratings do not distinguish imagination from reality

**DOI:** 10.1167/jov.24.5.13

**Published:** 2024-05-30

**Authors:** Nadine Dijkstra, Matan Mazor, Stephen M. Fleming

**Affiliations:** 1 Department of Imaging Neuroscience, University College London, London, UK; 2 All Souls College and Department of Experimental Psychology, University of Oxford, Oxford, UK; 3 Max Planck UCL Centre for Computational Psychiatry and Ageing Research, University College London, London, UK; 4 Department of Experimental Psychology, University College London, London, UK

**Keywords:** mental imagery, reality monitoring, metacognition, Bayesian modeling

## Abstract

Perceptual reality monitoring refers to the ability to distinguish internally triggered imagination from externally triggered reality. Such monitoring can take place at perceptual or cognitive levels—for example, in lucid dreaming, perceptual experience feels real but is accompanied by a cognitive insight that it is not real. We recently developed a paradigm to reveal perceptual reality monitoring errors during wakefulness in the general population, showing that imagined signals can be erroneously attributed to perception during a perceptual detection task. In the current study, we set out to investigate whether people have insight into perceptual reality monitoring errors by additionally measuring perceptual confidence. We used hierarchical Bayesian modeling of confidence criteria to characterize metacognitive insight into the effects of imagery on detection. Over two experiments, we found that confidence criteria moved in tandem with the decision criterion shift, indicating a failure of reality monitoring not only at a perceptual but also at a metacognitive level. These results further show that such failures have a perceptual rather than a decisional origin. Interestingly, offline queries at the end of the experiment revealed global, task-level insight, which was uncorrelated with local, trial-level insight as measured with confidence ratings. Taken together, our results demonstrate that confidence ratings do not distinguish imagination from reality during perceptual detection. Future research should further explore the different cognitive dimensions of insight into reality judgments and how they are related.

## Introduction

Perceptual reality monitoring—inferring whether sensory signals reflect reality or imagination—operates at perceptual and metacognitive levels (Dijkstra, Kok, & Fleming, 2022). Generally, perceptual experiences of reality correlate with metacognitive beliefs that such experiences indeed reflect reality. For example, during dreams or hallucinations, imagined content is perceived as real and also believed to reflect reality (Siclari et al., 2017; Zmigrod, Garrison, Carr, & Simons, 2016). However, there are cases where perception and metacognition of reality diverge. For example, in Charles Bonnet syndrome, a condition in which visual impairment is associated with the development of hallucinations, patients generally have insight into the unreality of their experiences (Menon, Rahman, Menon, & Dutton, 2003). Another example is lucid dreaming, where perceptual experience still “feels real” but people are aware that they are in fact dreaming (Baird, Mota-Rolim, & Dresler, 2019; Corlett, Canavan, Nahum, Appah, & Morgan, 2014; Konkoly et al., 2021).

One way to objectively characterize dissociations between perceptual and metacognitive processes is to ask how people evaluate confidence in their percepts (Fleming & Daw, 2017; Fleming & Lau, 2014). Metacognitive insight into effects of imagery on perception can then be measured as shifts in confidence: If participants *perceive* illusory objects when imagining and at the same time *know* they have this tendency, they might report seeing illusory objects but be less confident in these reports. If, on the other hand, they do not know they have this tendency, subjective confidence should exhibit the same qualitative effects as perceptual decisions.

More precisely, if confidence shifts in tandem with biases in perception, this indicates an absence of metacognitive insight. In line with this idea, recent studies of perceptual illusions (such as motion and color afterimages) have documented confidence shifts in tandem with idiosyncratic perceptual biases (Gallagher, Suddendorf, & Arnold, 2019; Mamassian & de Gardelle, 2022). Indeed, changes in confidence have been proposed as a diagnostic feature of truly perceptual (as opposed to decision-level) biases (Gallagher et al., 2019). In turn, recent computational models have suggested that perceptual confidence reflects a probability that one's response is self-consistent rather than objectively correct (Boundy-Singer, Ziemba, & Goris, 2022; Mamassian & de Gardelle, 2022). In contrast, other studies have documented cases in which participants’ confidence shows telltale signs of insight into a first-order decision bias, with so-called counterfactual confidence being used to update prior beliefs (Zylberberg, Wolpert, & Shadlen, 2018). Similarly, cases in which metacognitive sensitivity is greater than performance have also been documented (Scott, Dienes, Barrett, Bor, & Seth, 2014), indicating that some evidence may be accessible to confidence judgments that is not incorporated into perceptual decisions.

In this study, we aimed to characterize whether participants have insight into perceptual reality monitoring errors by investigating perceptual confidence in a scenario where both imagery and perception are at play. We have previously found that simultaneously imagining congruent stimuli during perceptual detection leads to an increase in presence responses, indicating that imagined signals are sometimes mistaken for perception (Dijkstra, Kok, & Fleming, 2021; Dijkstra, Mazor, Kok, & Fleming, 2021; Dijkstra & Fleming, 2023). Here, we investigated to what extent participants are metacognitively aware of these reality monitoring errors by additionally measuring confidence. We operationalize (lack of) insight here as the extent to which confidence shifts in tandem with imagery-induced biases in perception. Within signal detection theoretic models of confidence, such shifts can be quantified as the distance between decision criteria and confidence criteria—if such a distance remains invariant under shifts of decision criteria, this would reflect a lack of insight. To evaluate this hypothesis, we extended a hierarchical Bayesian model of confidence ratings (Fleming, 2017) to include a prior over the distances between decision and confidence criteria, enabling us to infer metacognitive insight about perceptual reality monitoring errors.

## Materials and Methods

This study was preregistered at https://osf.io/n39tm/. We initially planned for single-subject analyses, and after first data collection, we realized that a high number of participants did not have any false alarms or misses in one of the conditions. Therefore, we next tried to reduce this number by staircasing the decision-level criterion in a second data collection (updated preregistration). However, after further consideration, we eventually adopted a hierarchical model, which allowed us to relax these criteria and include all participants. Moreover, because the staircasing procedure was orthogonal to our main question, we decided to combine the two data sets for the final analyses to achieve maximum statistical power. To ensure the validity of our results, we replicated our findings in an independent sample in Experiment 2.

### Participants in Experiment 1

In total, 130 participants (half with the first staircasing procedure and half with the second) were recruited using Prolific (www.prolific.co) and completed the study online. Informed consent was obtained from each participant included in the study. The experiment took approximately 50 min to complete, and participants were paid £7.50 (£9 hourly rate, which is more than the preregistered hourly rate of £7.50 due to an update of the default within Prolific). All procedures were approved by the University College London ethics committee. Data from eight participants were not obtained due to technical issues. Participants were furthermore excluded if (a) their mean detection accuracy over conditions was below chance, (b) they answered the imagery check (see below) correctly on fewer than two blocks in any of the conditions, (c) they indicated in the debrief questions that they did not imagine the gratings as instructed, and (d) if, for a given detection response (yes/no), they used the exact same confidence rating in more than 90% of the trials. Six participants were moved due to below 55% detection accuracy, 13 due to too few correct imagery blocks, 1 due to indicating they had not imagined as instructed, and 0 due to too little variance in confidence ratings. In total, 102 participants (mean age = 30.4, *SD* = 8.5) were included in the final analyses.

### Experimental design and procedure in Experiment 1

**Figure 1.**
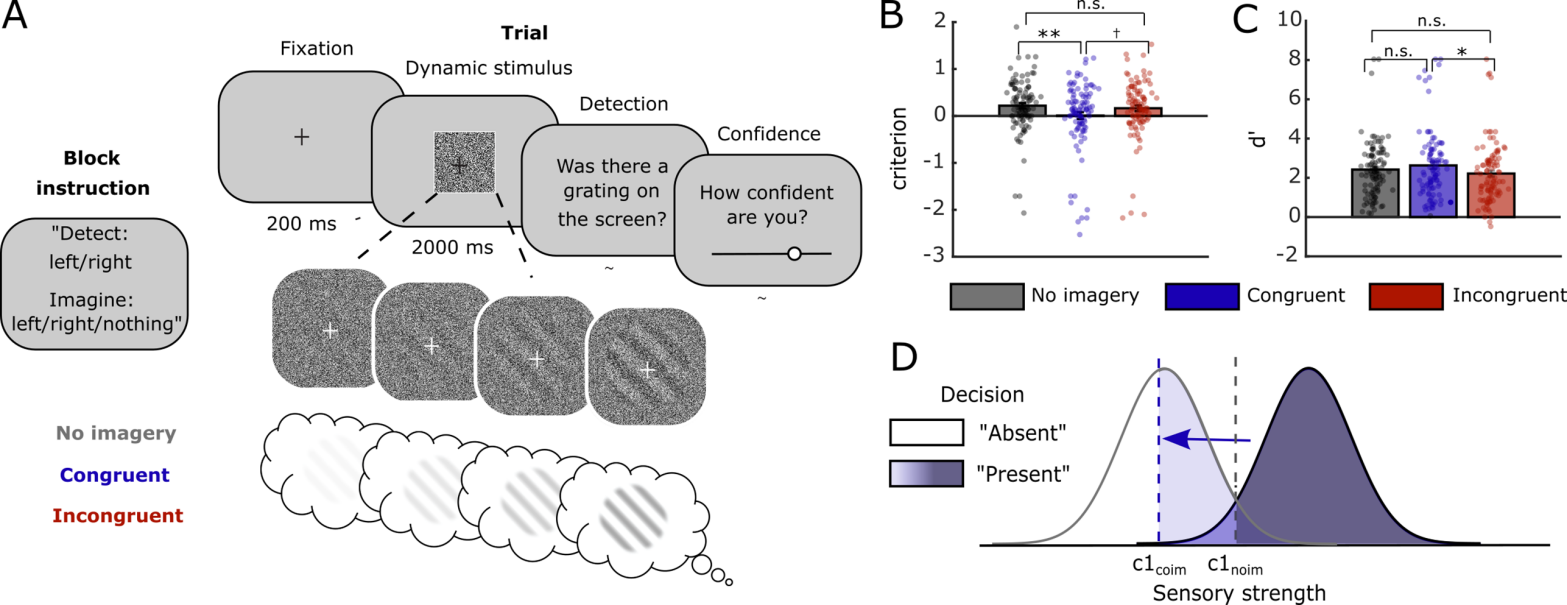
Experimental design, decision-level responses, and model. (**A**) Participants were instructed to detect oriented gratings in noise while simultaneously imagining the same grating (congruent), a grating perpendicular to the to-be-detected stimulus (incongruent), or nothing (no imagery). After each trial, participants indicated whether a stimulus had been presented on the screen and after that indicated the confidence in their answer from “complete guess” to “absolutely certain” by moving a slider with their mouse. (**B**) Decision criterion was significantly lower during congruent imagery compared to no-imagery and marginally lower during congruent imagery compared to incongruent imagery. There was no significant difference in criterion between incongruent imagery and no imagery. (**C**) In contrast, there was no effect of *d*′ during congruent imagery, but there was a significant decrease in *d*′ during incongruent imagery. (**D**) Signal detection theory (SDT) model of (congruent) imagery increasing perceptual presence responses by decreasing the decision-level criterion. Within SDT, a decrease in criterion is equivalent to an increase in the mean sensory strength of both the noise and signal distributions. *c*1_noim_ = first-order criterion during no imagery; *c*1_coim_ = first-order criterion during congruent imagery. *p < 0.05; **p < 0.005; †*p* < 0.06; n.s., *p* > 0.1.

To explain the concept of mental imagery to participants in a systematized way, they started the study by filling out a selection of the Vividness of Visual Imagery Questionnaire (VVIQ2; Marks, 1995). We chose to focus only on the “shop” part of the questions to save time and because this part of the questionnaire has been shown to lead to the highest vividness scores in the general population (Aphantasia Network; https://aphantasia.com). This part instructed the participant to “Think of a shop which you often go to. Consider the picture that comes before your mind's eye.” And then answer questions like “How vivid is the overall appearance of the shop from the opposite side of the road?” which participants have to rate from “no image at all, you only “know” that you are thinking of the object” to “perfectly clear and as vivid as real seeing” on a scale from 1 to 5. After the VVIQ2, participants practiced detecting gratings in dynamic noise (Figure 1) for six trials or until they had at least 75% accuracy.

After this, the staircasing procedure started. For the first data collection, we staircased the visibility of the grating only based on performance—aiming for a 70% detection accuracy. We staircased only one orientation to save time and because previous experiments showed that the threshold visibility values were comparable between the two orientations. The staircase contained 120 trials, and accuracy was calculated after every 10 trials. Visibility was increased if accuracy was below 65% and decreased if it was above 75% correct. After this, the concept of confidence was explained to participants and they practiced indicating confidence in their decision. Finally, participants practiced imagining the gratings in noise and rated their imagery vividness afterward on a scale from 1 to 5 for 10 trials per orientation before continuing to the main task. Analyzing these data revealed that this staircasing procedure was suboptimal, resulting in accuracies higher than the intended threshold (*M* = 83.6%, *SD* = 11.7% for the no-imagery condition) and leading to a low number of false alarms and misses.

For the second data collection, we therefore additionally aimed to staircase the criterion so that we would obtain sufficient number of misses and false alarms. Furthermore, we now included the confidence ratings already within the staircase to make it more comparable to the main task. Criterion was staircased by presenting prompts: If there were no misses in a mini-block of 16 trials, we presented, “Remember that sometimes noise might look like a grating and gratings are present on 50% of the trials”; conversely, if there were no false alarms, we presented “Remember that the gratings are hard to detect and there is a grating present on 50% of the trials.” This was repeated until the accuracy was between 50% and 100% and participants had at least one hit and one miss, or after five blocks of 16 staircase trials had passed, whichever came first. Finally, participants practiced imagining the gratings in noise. The resulting accuracies were slightly closer to our intended threshold but still higher than intended (*M* = 80.7%, *SD* = 13.5% for the no-imagery condition).

The main experimental design is shown in Figure 1. In order to avoid visual priming, trialwise cues were not delivered, and instead the different conditions were implemented in a blockwise fashion such that during the entire block, participants detected one specific orientation and imagined one specific orientation. At the onset of each experimental block, the participant was instructed which orientation to imagine and which orientation to detect. To ensure that participants accurately followed the imagination instructions, after each block, we asked participants which orientation they had imagined. We only analyzed data of blocks that were answered correctly and of participants who answered this imagery check correctly in most blocks (see above). There were 12 experimental blocks in total, 4 per imagery condition, each consisting of 24 trials. The order of the blocks was randomized within each participant. Participants used the “F” and “G” keys with their left hand to perform the detection task and the mouse with their right hand to indicate their confidence on a continuous scale. Response-key mappings—which key, “F” or “G,” corresponds to presence and which key to absence—were randomized over participants. To ensure participants properly indicated their confidence, they had to move the confidence slider on each trial, at least a small amount, before the experiment continued. At the end of the experiment, we asked the following open-ended questions: (a) the participant's age; (b) if they imagined the gratings as instructed; (c) if they thought that imagining the grating influenced whether they saw one and, if yes, how (only for the second data collection); and (d) any if they had any further comments.

### Stimuli

The stimuli were generated in MATLAB (version R2018b) and consisted of sinusoidal gratings with a spatial frequency of 0.7, tilted at an orientation of 45^o^ or 135^o^, masked with an annulus and embedded in white noise (Figure 1). The visibility of the stimuli was manipulated by changing the probability that a given pixel was replaced by a random value. For each orientation separately, stimulus images of 50 visibility levels were generated. Visibility levels ranged from 0% to 14%, distributed equally in log space. For the absence trials, 20 images of pure white noise were generated. The main experiment was programmed in JavaScript using jsPsych (de Leeuw, 2015). During stimulus-present trials, 20 stimulus images ranging from zero visibility to threshold level were presented over the course of 2 s, giving the impression that the stimulus was gradually ramping up. This ramping up was done to mimic the gradual nature of mental image generation (Perky, 1910). During stimulus-absent trials, 20 noise images were presented in random order.

### Participants in Experiment 2

We performed a power calculation based on the results from Experiment 1 to determine the number of participants for Experiment 2. Assuming an effect size of 0.32, 103 participants would be required to reach a power of 90% with a two-sided alpha level of 0.05. Taking into account dropout, 130 participants gave informed consent and completed the study online. Data recruitment, collection, and exclusion criteria were identical to the first experiment. Data from three participants were not collected due to technical issues, eight participants were removed due to below 55% detection accuracy, six due to too few correct imagery blocks, zero due to indicating they had not imagined as instructed, and two due to too little variance in confidence ratings. In total, 111 participants (mean age = 33.16, *SD* = 9.89) were included in the final analyses.

### Experimental design and procedure in Experiment 2

The design and experiment were identical to both data collections of Experiment 1 except that the staircasing procedure was now a mix between the two. Specifically, in Experiment 2, the confidence rating was included in the staircasing procedure and the visibility was only staircased based on accuracy. The visibility first quickly went down to a level that corresponded to approximately threshold performance in the previous sample and was then fine-tuned depending on the participant's response: going up if accuracy was below 60% and going down if it was above 80%. This led to an accuracy of 75.13% (*SD* = 10.3%) for the no imagery condition, closer to the intended threshold than the values obtained with the previous two staircase procedures. At the end of the main experiment, a global insight question was asked as follows:

On some blocks you imagined [the same]/[a different] grating as the one you had to detect. Relative to the blocks where you didn't have to imagine anything, how did this affect your tendency to say there was a grating on the screen?

Relative to not imagining anything, imagining [the same]/[a different] grating . . .

The answer was indicated using a slider that ranged from “made me much *less* like to report seeing a grating” to “made me much *more* likely to report seeing a grating” with “had no effect” in the middle.

### Data analyses

We analyzed the first-order decision responses using standard signal detection theory (Green & Swets, 1966). Detection sensitivity (*d*′) and criterion (c) were calculated separately for the imagery and no-imagery trials as follows:

$$\begin{array}{c} d^{'}=z\left(H\right)-z\left(FA\right) \end{array}$$

$$\begin{array}{c} c=-0.5\times \left[z\left(H\right)+z\left(FA\right)\right] \end{array}$$

where *z* indicates the inverse of the cumulative normal distribution, *H* is the hit rate (the proportion of present trials for which the participant reported presence), and *FA* is the false alarm rate (the proportion of absent trials for which the participant reported presence). Detection sensitivity *d*′ is a measure of detection performance, with greater values indicating better performance. Criterion *c* is a measure of participant’s bias toward responding “yes” (present) or “no” (absent), irrespective of whether a stimulus is present or not. Greater values of *c* indicate a more conservative criterion, indicating a greater tendency toward reporting absence. Hit rates of 1 or false alarm rates of 0 lead to biased estimations of *d*′ and c. To correct for this, in those cases of extreme values, we added a count of 0.5 to the relevant cell (Hautus, 1995).

Confidence ratings were first analyzed with a repeated-measures with response (“absent” vs “present”) × condition (no imagery vs. congruent vs. incongruent) as within-subject variables. Next, we extended the hierarchical meta-*d*′ model (Fleming, 2017) to allow inference on metacognitive insight into perceptual reality monitoring errors. In contrast to standard frequentist approaches, the Bayesian framework allowed us to quantify evidence in favor of the *absence* of metacognitive insight. Prior to fitting the model, we split the confidence ratings of each subject and each condition into low and high by first *z*-scoring the data and counting all ratings above 0 as high and below 0 as low.

Within the original HMeta-*d*′ model, the group-level parameter of interest is M-ratio *M*, the ratio between second-order meta- *d*′ and first-order decision *d*′:

$$M=\frac{\mathrm{meta}-d^{'}}{d^{'}}$$

where meta-*d*′ is calculated by estimating what the first-order *d*′ would be based on the observed confidence ratings alone and *d*′ is a fixed parameter directly calculated from the decision data (Fleming, 2017; Maniscalco & Lau, 2012). An *M* below 1 indicates that there is information loss when going from decision to confidence rating, whereas an *M* above 1 indicates that additional information is incorporated at the confidence rating stage.

**Figure 2.**
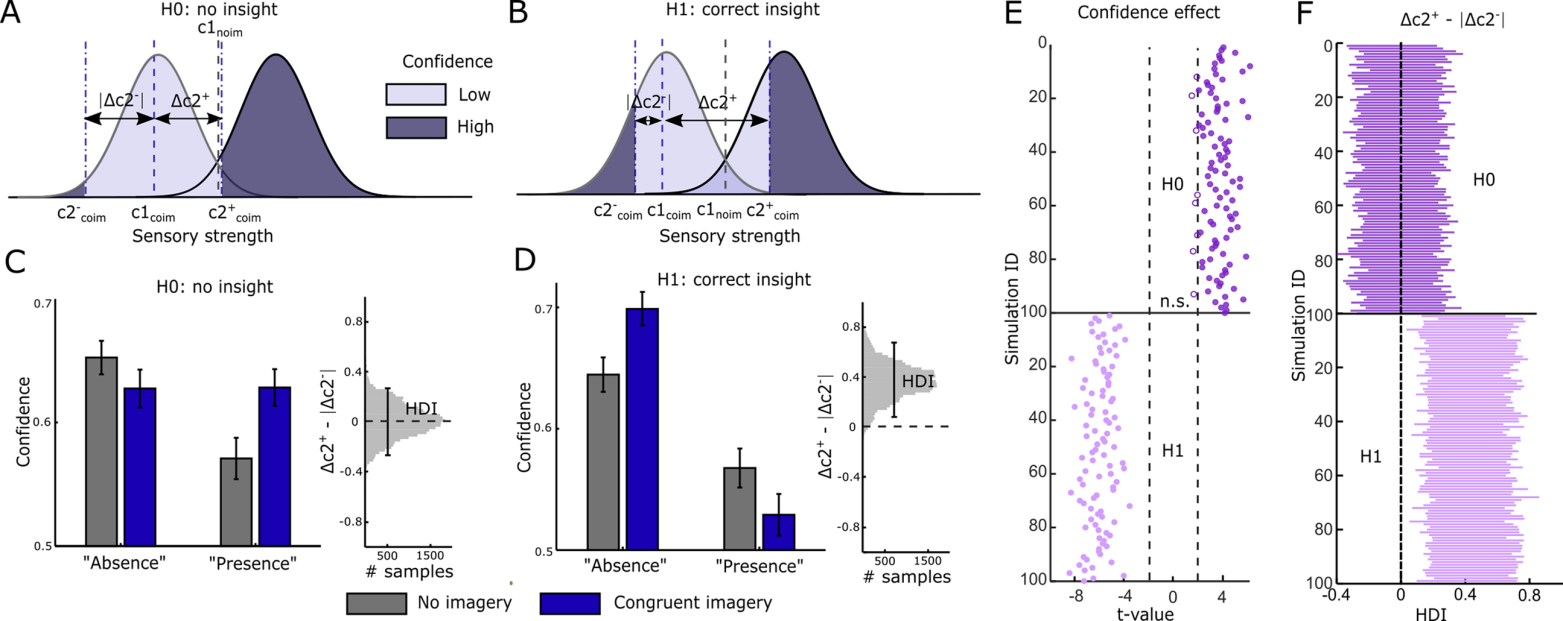
Modeling insight into perceptual reality monitoring. (**A**) Illustration of shift in confidence criteria compared to first-order criterion. If participants have no insight into their first-order criterion shift, we would expect confidence criteria to move with the decision-level criterion shift. coim = congruent imagery; *c*1 = first-order criterion; *c*2^−^ = negative second-order (metacognitive) criterion; *c*2^+^ = positive second-order criterion; Δ*c*2+/− = c2 relative to c1 (i.e., *c*2–*c*1). (**B**) In contrast, if participants have insight into the fact that imagery increases perceptual presence, their confidence criteria would remain closer to the no-imagery criterion. (**C**) Simulated confidence ratings under a no-insight model. Left: compared to no imagery, imagery leads to lower confidence in absence responses and higher confidence in presence responses. Right: asymmetry between positive and negative confidence criteria, relative to the position of the first-order criterion (Δ*c*2+ – |Δ*c*2–|). The black line indicates the HDI representing the 95% most credible values of the posterior. The HDI is centered around 0, indicating symmetrical positive and negative Δ*c*2 values and therefore a symmetrical shift in confidence criteria relative to the Type 1 criterion effect. (**D**) Simulated confidence ratings under a full-insight model. Left: imagery leads to higher confidence in absence responses and lower confidence in presence responses. Right: there is a positive asymmetry between positive and negative Δ*c*2 values, indicating that confidence criteria stay closer to what would be expected under no imagery, in line with insight into the effect of imagery on perception. (**E**) The confidence effect, operationalized as the interaction between condition and response on confidence ratings, for repeated independent simulations under no insight (H0; top, dark purple) and full insight (H1; bottom, light purple), with other parameter values drawn from distributions consistent with the empirical data. Positive *t*-values reflect a decrease in confidence for absence and an increase for presence for imagery versus no imagery, whereas negative *t*-values reflect the opposite. Filled circles have a *p* value <0.05 (uncorrected). (**F**) HDIs of the asymmetry between positive and negative confidence criteria for repeated simulations under both the no-insight model (H0; top, dark purple) and the full-insight model (H1; bottom, light purple).

**Figure 3.**
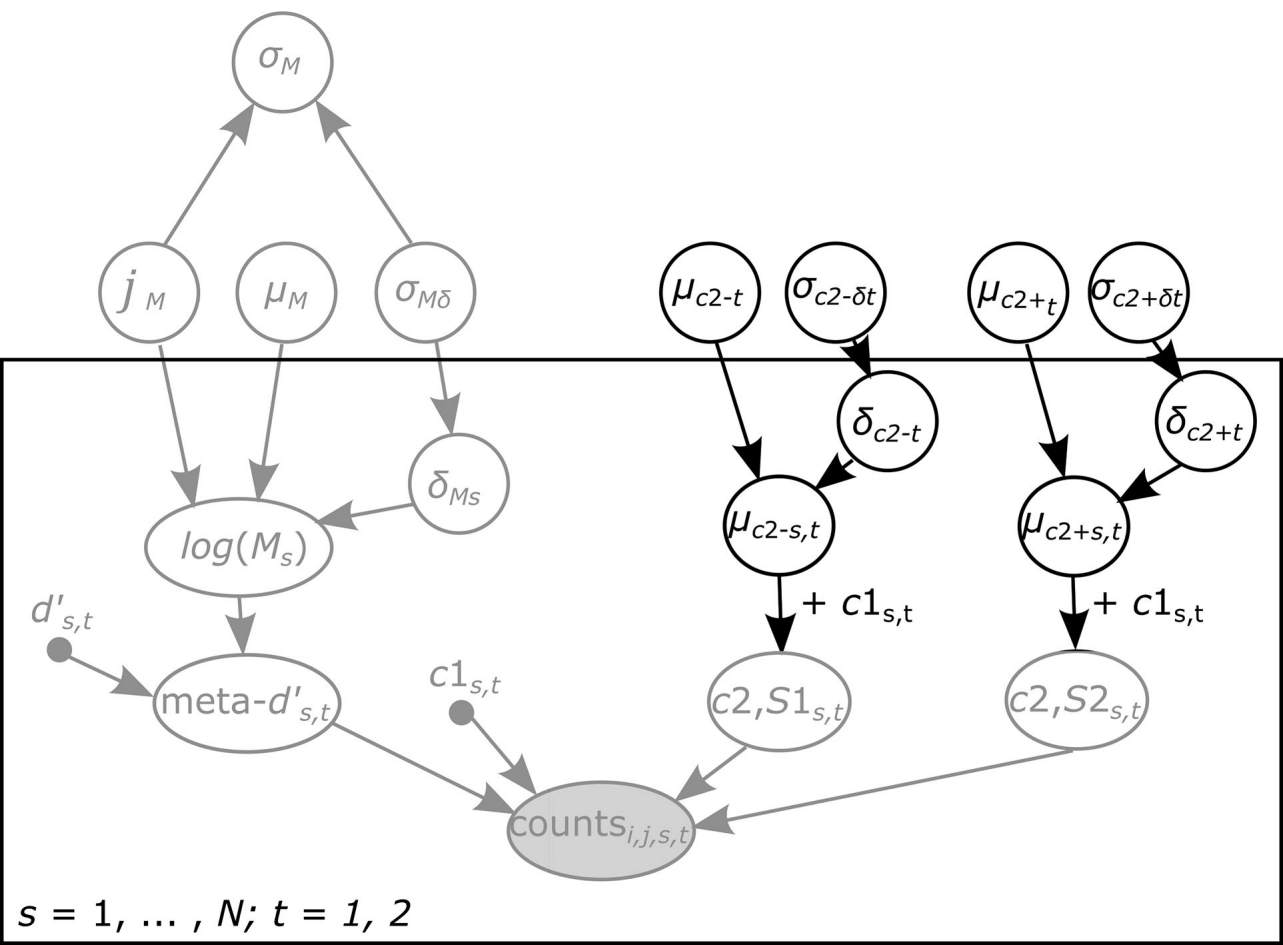
Current extension of the HMeta-*d*′ model. Probabilistic graphical model for estimating metacognitive insight into criterion shifts. A full description of the original HMeta-*d*′ model, here indicated in light gray, can be found in Fleming (2017). In short, the model aims to estimate trial counts (counts) per confidence rating bin conditional on both the stimulus category (S1, i.e., presence and S2, i.e., absence) and the response (S1, i.e., “present” and S2, i.e., “absent”). Given a particular setting of the parameters, the model specifies a multinomial probability distribution *P*(*conf* = *y* |*stim* = *i*,  *resp* = *j*) over observed confidence response counts. We extended the model to include group-level priors (mean and variance) over the difference between decision *c*1 and confidence *c*2 criteria, separately for negative (absence) *c*2– (µ*_c_*_2-t_ and σ*_c_*_2-t_) and positive (presence) *c*2+ (µ_c2+t_ and σ_c2+t_) responses. Group-level variance is translated into subject-specific precision δ_*c*2 − *t*_ and δ_*c*2 + *t*_. Point estimates for type 1 *d*′ and criterion are represented as black dots. All parameters in unfilled circles are free parameters estimated by the model. The box encloses participant-level parameters subscripted with *s*, whereas parameters outside the box represent group-level parameters. Condition specific (no-imagery vs. imagery) parameters are subscripted with *t*. We employ the scheme suggested by Matzke et al. (2014), such that the mean and variance of *log*(*Ms*) are scaled by a redundant multiplicative parameter ξ*M*. The posterior on σ*M* can then be recovered by adjusting for the influence of this additional random component.

Here, in contrast, we were interested in metacognitive insight into changes in decision criterion *c* rather than performance *d*′. Within the original HMeta-*d*′ model, the absolute distances between the decision criterion and the negative and positive confidence criteria are modeled as being symmetric (the priors for both positive and negative criteria are shared). This means that a shift in the decision criterion leads to an accompanying (symmetric) shift in the confidence criteria under the default priors (cf. Figure 2A vs. Figure 2B). Here, in contrast, in order to allow for independent shifts in insight, we included separate task-specific group-level priors over the negative *c*2– and positive *c*2+ confidence criteria (µ*_c_*_2-t_, σ*_c_*_2-t_ and µ*_c_*_2+t_, σ*_c_*_2+t_ in Figure 3). Furthermore, we modeled second-order criteria relative to the first-order criterion to allow for a direct comparison between the positive and negative *c*2 (Figure 3). Asymmetries between positive and negative *c*2 distances would indicate some form of metacognitive insight (cf. Figure 2).

To determine what kind of effects we would expect to find, we simulated confidence ratings under both a no-insight (H0) and a full-insight (H1) model. We based the group Type 1 parameters (*d*′, *c*1 no imagery, *c*1 imagery) on the data and set the group Type 2 criteria for the imagery condition separately for the no-insight and full-insight model as follows:

**Table:** 

Parameter	*c*1	*c*2 −	*c*2 +
**No** **imagery (for both models)**	0.2	−0.6	1
**Imagery (no** **-** **insight model)**	0	−0.8	0.8
**Imagery (full** **-** **insight model)**	0	−0.6	1

The group Type 1 *d*′ was set to 2.5 and the group M-ratio—a measure of metacognitive sensitivity—was set to 0.8. For each condition, we simulated 100 participants with 96 trials each. For each participant, parameter values were drawn from a Gaussian distribution with a mean set to the group values listed above and a sigma of 0.2 for Type 1 parameters and 0.5 for Type 2 parameters. We next used the function “metad_sim.m” from the HMeta-*d*′ toolbox (Fleming, 2017) to calculate the probability of different trial types (low and high confidence false alarms, hits, misses, and correct rejections) under a Type 2 signal detection theoretic model with these parameters. Confidence ratings for absence responses were calculated as the proportion of high-confidence correct rejections and high-confidence misses divided by the total proportion of correct rejections and misses. Confidence ratings for presence responses were calculated as the proportion of high-confidence hits and high-confidence false alarms divided by the total proportion of hits and false alarms. We next applied both frequentist as well as hierarchical Bayesian statistics on the simulated data to determine the expected results under the two models (see Figure 2 and main text for more details).

## Mistaking imagination for reality during perceptual detection

In a first experiment, 102 participants performed a perceptual detection task on gratings that gradually appeared within dynamic noise while simultaneously imagining either the same grating (congruent) or a grating orthogonal to the one they were detecting (incongruent) or nothing (no imagery; Figure 1A). In line with previous findings, we observed a significant decrease in decision criterion (that is, an increased tendency to report stimulus presence) specifically for congruent imagery (*M* = 0.01, *SD* = 0.73) compared to no imagery (*M* = 0.22, *SD* = 0.57, *t*(101) = 3.10, *p* = 0.0025, *d* = 0.32, 95% *CI* difference = 0.07 to 0.34; Figure 1B). While the criterion for congruent imagery was numerically lower than that for incongruent imagery (*M* = 0.16, *SD* = 0.61), this difference did not reach significance (*t*(101) = 1.98, *p* = 0.0502, *d* = 0.23, 95% *CI* difference = −0.0002 to 0.31). Finally, there was no significant difference in criterion between incongruent imagery and no imagery (*t*(101) = −0.96, *p* = 0.341, *d* = −0.09, 95% *CI* difference = −0.16 to 0.06). Instead, and consistent with previous findings, there was a significant decrease in *d*′ during incongruent imagery (*M* = 2.22, *SD* = 1.54) compared to congruent imagery (*M* = 2.63, *SD* = 1.76; *t*(101) = −2.50, *p* = 0.014, *d* = −0.25, 95% *CI* difference = −0.74 to −0.09). Together, these results demonstrate that participants were more likely to indicate perceptual presence when simultaneously imagining the same stimuli while incongruent imagery merely decreased performance (Figure 1D). These results are in line with previous studies and are interpreted to reflect perceptual reality monitoring errors: Participants are more likely to say there was a grating on the screen when the same stimuli were both imagined and perceived (Dijkstra et al., 2021; Dijkstra et al., 2021; Dijkstra & Fleming, 2023).

## Quantifying insight into perceptual reality errors

We next aimed to characterize insight into these reality monitoring errors using a signal detection theoretic model of metacognition. As noted above, within a signal detection framework, congruent imagery leads to a more liberal decision criterion and more “presence” responses, which we interpret as being the result of imagery adding signal strength to both the noise and signal distribution. Subjects who are metacognitively aware of this shift should be less confident in their “presence” responses when they are imagining the target stimulus, because a liberal criterion means they are more likely to commit a false alarm. Similarly, they should be more confident in their “absence” responses when imagining the target stimulus, because a liberal criterion means they are less likely to miss presented stimuli. In contrast, if subjects have no metacognitive insight into the effects of imagery on their perceptual presence responses, they should be more confident in their “presence” responses and less confident in their “absence” responses, as their confidence ratings should follow their perceptual bias towards reporting “presence.”

These effects can be quantified by tracking the position of their metacognitive (confidence) criteria: the cutoff points at which participants rate their decision as high versus low confidence (Figures 2A, 2B). If participants have no insight into the influence of imagery on their responses, confidence criteria should shift in tandem with the decision-level criterion, and the distance between the confidence criteria and the Type 1 criterion should be symmetric for positive and negative values (Figure 2A). In contrast, if participants are aware that imagery increases perceptual presence, confidence criteria should remain closer to the no-imagery condition, creating an asymmetry in distances between the positive and negative confidence criteria and the Type 1 criterion (Figure 2B).

In order to arbitrate between these two hypotheses, we simulated what the data would look like under the two models (H0: no insight and H1: correct insight) using similar type 1 *d*′ and *c* parameter values to those found in the empirical data (Figure 1). In line with intuition, under a no-insight model, congruent imagery is associated with a decrease in confidence for “absence” and an increase in confidence for “presence” compared to no imagery (Figure 2C, left). In contrast, correct insight shows exactly the opposite pattern with an increase in confidence for “absence” and a decrease in confidence for “presence” (Figure 2D, left).

Another, more model-based way to measure the level of insight is by estimating the asymmetry between the positive and negative confidence criteria relative to the first-order decision criterion: If there is no insight, the confidence criteria will move in tandem with the decision criteria, leading to symmetrical distances between *c*1 and *c*2^+^ and between *c*1 and *c*2^−^ (Figure 2A). In contrast, in the case of a correct insight, the confidence criteria will remain close to the no-imagery case despite the decrease in the first-order criterion. This will result in a larger distance between *c*2^+^ and *c*1 than between *c*2^−^ and *c*1 (Figure 2B).

To directly characterize this asymmetry in confidence criteria, we extended the hierarchical meta-*d*′ model (Fleming, 2017; Maniscalco & Lau, 2012). Specifically, we modeled the second-order (metacognitive) criteria on confidence as free parameters and allowed for asymmetries between the distances from the decision criterion to the negative (“absence”) and positive (“presence”) confidence criteria. Applying this analysis to our simulated data indeed showed that no insight was associated with symmetric confidence criteria; that is, the highest-density interval (HDI) of the difference between positive and negative confidence and decision criteria was centered around 0 (Figure 2C, right). In contrast, correct insight was associated with a larger positive confidence criteria distance with the HDI of the difference exceeding 0 (Figure 2D, right).

We next repeated these simulations 100 times for each model to estimate the reliability of each of these effects. These simulations revealed that the confidence effect, defined as a *t*-test on the interaction between condition (imagery vs. no imagery) and response (presence vs. absence), was always positive under the no-insight model and always negative under the correct insight model (Figure 2E). However, the confidence interaction effect under the no-insight model appeared to be statistically less robust with more *t*-values closer to the significance threshold. The HDI of the difference in confidence criteria was always centered around 0 for no insight and always above 0 for correct insight (Figure 2F). To characterize metacognitive insight in our empirical data, we estimated both the confidence effect using classical statistics as well as the asymmetry of the confidence criteria using our extension of the hierarchical meta-*d*′ model.

## Confidence ratings reveal absence of insight into perceptual reality errors

After successful validation of our analysis pipeline, we next turned to our empirical confidence ratings. Prior to applying our analysis pipeline to investigate insight into reality monitoring errors, we first checked whether there were any differences in metacognitive efficiency between the conditions using the standard hierarchical meta-*d*′ model (Fleming, 2017). This analysis revealed no significant differences in metacognitive efficiency (meta-*d*′/*d*′) between no imagery (95% HDI = 0.53 to 0.67), congruent imagery (HDI = 0.47 to 0.65), and incongruent imagery (HDI = 0.48 to 0.65), indicating that metacognitive sensitivity was similar between the conditions (Supplementary Fig. S1). We next investigated the effects of condition on both confidence and the asymmetry in confidence criteria to characterize metacognitive insight (Figure 2).

**Figure 4.**
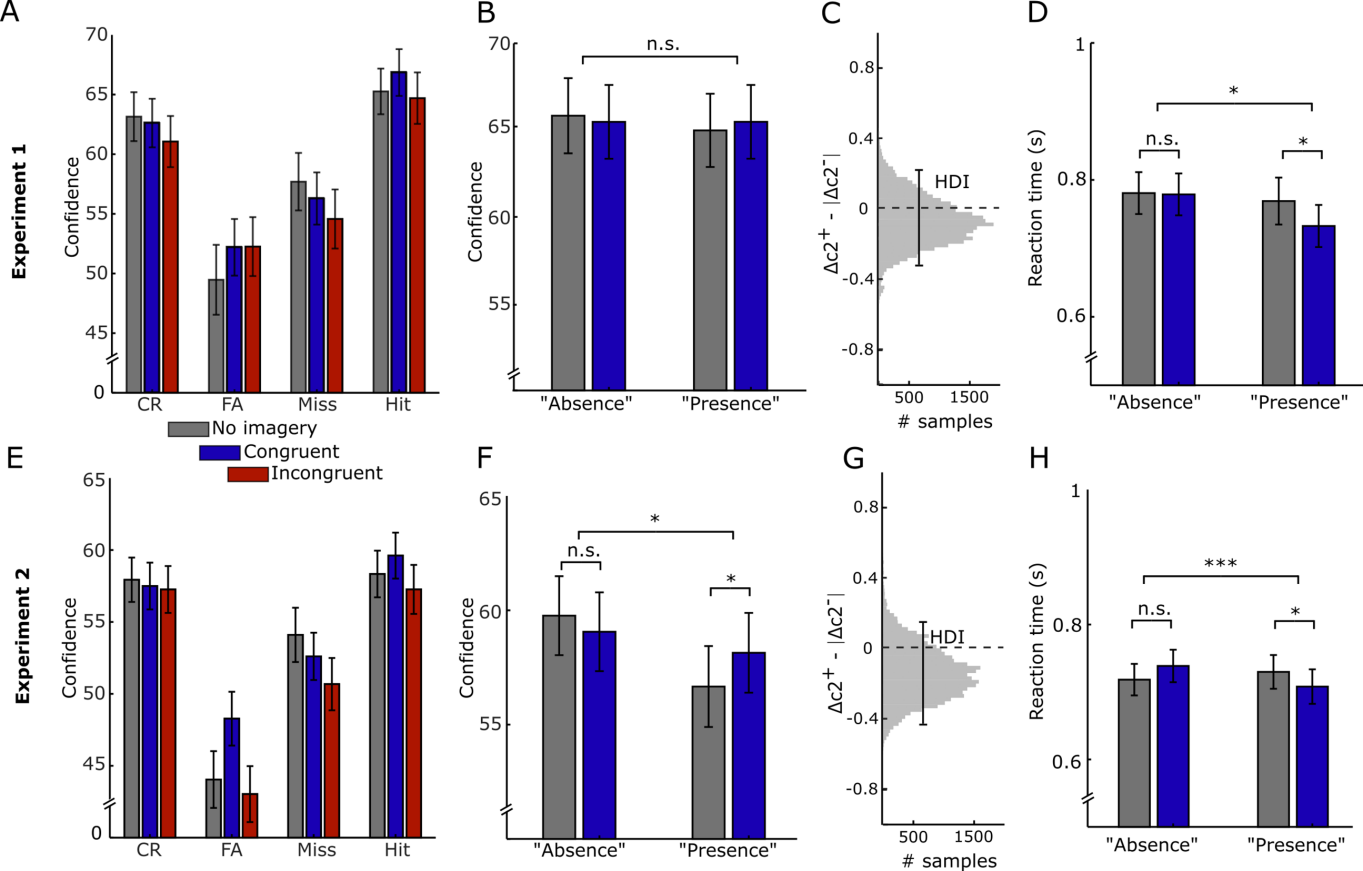
Metacognitive insight during source mixing. (**A**) Confidence ratings per condition and trial type for Experiment 1 (CR = correct rejection; FA = false alarm). Only data for participants with all trial types in all conditions are shown (*N* = 59). (**B**) Confidence ratings per response category for no-imagery and congruent imagery conditions, as in Figures 2C, 2D, included data from all participants. (**C**) Asymmetry between positive and negative confidence criteria*,* relative to the position of the first-order criterion. Positive values indicate more insight (cf. Figures 2C, 2D). (**D**) Reaction times per response for no-imagery and congruent imagery conditions. (**E**–**H**) Same as **A**–**D** for Experiment 2. *p < 0.05; ***p < 0.0005.

We first ran a repeated-measures analysis of variance (ANOVA) test on the confidence ratings from all conditions, including only data from participants with misses and false alarms in all conditions (*N* = 59). This revealed no significant effects of condition (Figure 4A). Furthermore, directly comparing confidence in presence and absence responses, irrespective of input, between no imagery to congruent imagery qualitatively followed the no-insight pattern, with higher confidence for presence responses and lower confidence for absence responses during congruent imagery (Figures 4A, 4B), but there was no significant interaction between condition and response (*t*(101) = 0.94, *p* = 0.35). Fitting our extension of the hierarchical meta-*d*′ model to our data provided a good fit (Supplementary Fig. S2). The results showed that the asymmetry between the negative and positive confidence criteria was centered around 0, in line with no insight (Figure 4C; *M* = −0.07, 95% HDI = −0.33 to 0.22). Moreover, the relative confidence criteria were comparable between the imagery and no-imagery conditions, showing that they indeed shifted in tandem with the perceptual criterion shift (95% HDI Δ*c*2 + _no_imagery_^−^Δ*c*2 + _congruent_imagery_ = −0.3 to 0.28 and 95% HDI Δ*c*2 – _no_imagery_^−^Δ*c*2 – _congruent_imagery_ = −0.29 to 0.26). Finally, given that reaction time often (negatively) correlates with confidence (Rahnev et al., 2020), we performed an exploratory analysis to investigate the effects of condition on reaction time. There was a significant interaction between condition and response (*t*(101) = −2.07, *p* = 0.041, *d* = 0.26, *CI* = −0.067 to −0.001; Figure 4D), with faster presence responses during congruent imagery (*t*(101) = 2.17, *p* = 0.032, *d* = 0.22; *CI* = 0.282–0.696).

Given that first-order performance was higher than anticipated in Experiment 1, potentially due to our staircasing procedure, and that some results were unclear, we decided to repeat the experiment in an independent sample with a slightly different staircasing procedure (see Materials and Methods for more details) to ensure that our findings are replicable. The experimental procedure was mostly identical to the first but also included global insight questions at the end (see section below). After exclusion, data from 111 participants were analyzed. We first again replicated the decision-level effect, showing a decrease in criterion for congruent imagery (*M* = 0.23, *SD* = 0.50) compared to no imagery (*M* = 0.35, *SD* = 0.53, *t*(110) = 2.48, *p* = 0.015, *d* = 0.22, 95% *CI* difference = 0.02–0.21) but not for incongruent imagery (*M* = 0.32, *SD* = 0.56, *t*(110) = 0.813, *p* = 0.42, *d* = 0.06, 95% *CI* difference = −0.04 to 0.10). In contrast, there was a significant decrease in *d*′ for incongruent imagery (*M* = 1.56, *SD* = 1.25), compared to no imagery (*M* = 1.72, *SD* = 1, *t*(110) = 2.16, *p* = 0.033, *d* = −0.14, 95% *CI* difference = 0.01–0.32), but not for congruent imagery (*M* = 1.75, *SD* = 1.27, *t*(110) = −0.27, *p* = 0.79, *d* = 0.02, 95% *CI* difference = −0.2 to 0.15). These results again show that participants were more likely to indicate perceptual presence when imagining the same stimulus, whereas imagining a different stimulus merely decreased performance. Finally, similar to Experiment 1, there were no significant differences in metacognitive efficiency between no imagery (HDI = 0.44 to 0.59), congruent imagery (HDI = 0.37 to 0.55), and incongruent imagery (HDI = 0.45 to 0.61; Supplementary Fig. S1).

We next again first ran a repeated-measures ANOVA on the confidence ratings from all conditions, including only data from participants with misses and false alarms in all conditions (*N* = 59). This time, we did find a significant main effect of condition (*F*(182, 2) = 6.64, *p* = 0.002, *η*_p_^2^ = 0.068). Post hoc tests revealed that this effect was driven by a decrease in confidence in the incongruent imagery condition (*M* = 52.1, *SD* = 15.2) compared to both no imagery (*M* = 53.5, *SD* = 15.5, *t*(91) = 2.28, *p* = 0.79, *d* = 0.02, 95% *CI* difference = −0.2 to 0.15) and congruent imagery (*M* = 1.75, *SD* = 1.27, *t*(110) = −0.27, *p* = 0.79, *d* = 0.02, 95% *CI* difference = −0.2 to 0.15). There was no significant difference between congruent imagery and no imagery in confidence ratings (*t*(91) = 1.322, *p* = 0.188, *d* = −0.138, 95% *CI* difference = −0.25 to 0.74). This decrease in confidence during the incongruent condition presumably reflected the decrease in performance in that condition. Interestingly, there was now also a significant interaction between condition and response (Huyn–Feldt corrected *F*(172.48, 1.9) = 3.937, *p* = 0.023, *η*_p_^2^ = 0.041). Post hoc analyses revealed that confidence of presence responses, not absence responses, was higher for congruent imagery (*M* = 53.94, *SD* = 17.2) compared to no imagery (*M* = 51.19, *SD* = 17.83, *t*(91) = 2.71, *p* = 0.008, *CI* = 0.73–4.78, *d* = 0.16) and incongruent imagery (*M* = 50.15, *SD* = 17.9, *t*(91) = 4.3, *p* = 0.0004, *CI* = 2.04–5.55, *d* = 0.22).

In line with this, directly comparing confidence for presence and absence responses irrespective of input between only no imagery and congruent imagery revealed a significant interaction between condition and response on confidence ratings (*t*(110) = 2.302, *p* = 0.023, *d* = −0.30, *CI* = 0.30 to 4.05). Post hoc paired *t*-tests revealed that confidence of presence responses, not absence responses, was higher for congruent imagery compared to no imagery (*t*(110) = −2.37, *p* = 0.019, *d* = −0.23, *CI* = −2.71 to −0.24; Figures 4E, 4F), in line with no insight (cf. Figure 2C). We next quantified metacognitive insight using our extension of the hierarchical meta-*d*′ model. In line with the results from Experiment 1, we again found that the HDI was centered around 0, indicating that the confidence criteria moved along with the decision-level criterion, in line with no insight (Figure 4G, *M* = −0.14, 95% HDI = −0.44 to 0.15). Furthermore, the relative confidence criteria were again comparable between the imagery and no-imagery conditions (95% HDI Δ*c*2 + _no_imagery_^−^Δ*c*2 + _congruent_imagery_ = −0.26 to 0.27 and 95% HDI Δ*c*2 – _no_imagery_^−^Δ*c*2 – _congruent_imagery_ = −0.26 to 0.33). Finally, there was also a significant interaction between condition and response on reaction time (*t*(110) = −3.65, *p* = 0.004, *d* = 0.37, *CI* = −0.065 to −0.019), with faster presence responses during congruent imagery compared to no imagery (*t*(110) = 2.14, *p* = 0.035, *d* = 0.20, *CI* = 0.002 to 0.042), again in line with no insight.

Taken together, even though the results from the frequentist analyses of the confidence ratings were inconclusive, the qualitative pattern of results over the two experiments is in line with a no-insight model (cf. Figure 2C).

## Postexperiment queries reveal global insight into perceptual reality monitoring errors

Taken together, our results suggest that people do not have insight into the influence mental imagery has on their perception at the level of individual trials. However, one intriguing possibility is that they do have insight at a more global level but that this awareness is not incorporated into local within-task confidence ratings. Dissociations between local and global metacognition have previously been reported in relation to several dimensions of mental ill health (Bhome et al., 2022; Seow, Rouault, Gillan, & Fleming, 2021) as well as in normal aging (McWilliams, Bibby, Steinbeis, David, & Fleming, 2023).

**Figure 5.**
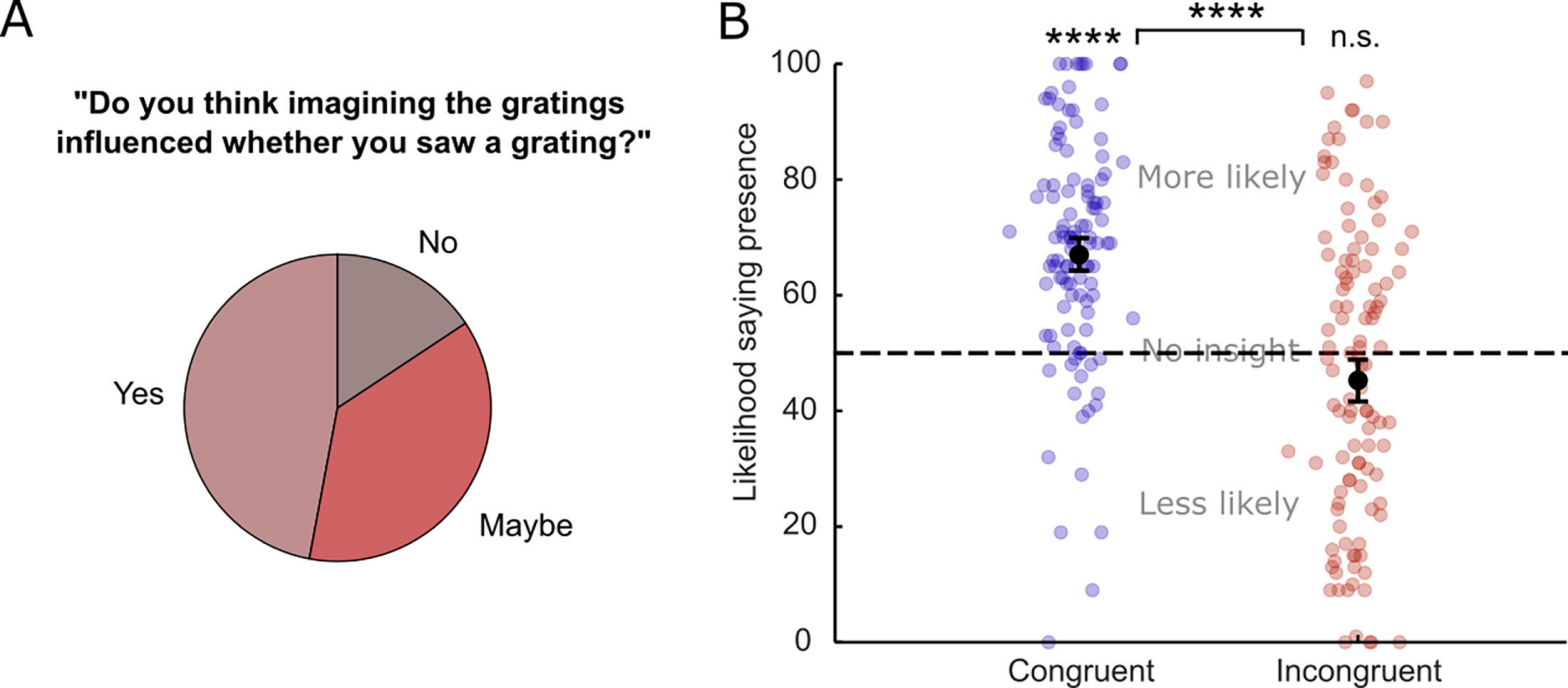
Postexperiment queries indicate insight. (**A**) Proportion of participants indicating that they thought imagery influenced their perceptual response after Experiment 1. *N* = 51. (**B**) Responses to the question whether imagery made participants more or less likely to indicate perceptual presence, separate for congruent (blue) and incongruent (red) conditions, after Experiment 2. Dots represent individual participants. ****p < 0.0001; n.s. = non-significant.

To investigate this possibility in the context of our study, we included global insight questions at the end of both experiments. At the end of Experiment 1, we asked the open-ended question, “Do you think imagining the gratings influenced whether you saw a grating?” Of the 51 participants who answered this question, 24 (47.1%) thought that imagery did have an influence, 19 (37.2%) were unsure, and only 8 (15.7%) thought that imagery did not influence was they saw (Figure 5A). Due to the qualitative nature of this question and the fact that only a small number of participants answered it, we were unable to link global insight to local insight in Experiment 1.

To address this, after the second experiment, we interrogated global insight using a structured questionnaire. We asked participants to indicate how they thought imagining the gratings influenced their detection responses relative to not imagining by moving a slider ranging from 0 = “made me less likely to say I saw a grating” to 100 = “made me more likely to say I saw a grating” with the center of the scale indicating 50 = “no effect.” This question was asked in relation to imagining the same grating (congruent) or imagining a different grating (incongruent).

Global insight ratings indicated that, overall, participants accurately indicated that congruent imagery made it more likely for them to report presence compared to no imagery (*M* = 68.14, *SD* = 19.79, *t*(110) = 9.65, *p* < 0.0001, *CI* = 64.41–71.86, *d* = 0.92), whereas for the incongruent imagery, the ratings were not significantly different from the center, “no effect” point (*M* = 46.63, *SD* = 25.92, *t*(110) = −1.37, *p* = 0.17, *CI* = 41.75–51.51, *d* = 0.13). This suggests that participants did have global insight into the effect of imagery on perceptual presence responses. However, there was no significant correlation between the extent to which participants believed imagery increased their presence responses and how much it actually did, measured by the decision-level criterion shift (*r* = 0.12, *p* = 0.2). Furthermore, there were no significant correlations between global insight and local confidence ratings in any of the conditions (all absolute *r*s < 0.15, all *p*s > 0.11). These results suggest that the insight judgment at the end of the experiment might have relied on different cognitive mechanisms than the decisions and confidence ratings elicited during the perceptual detection task.

## Discussion

In this study, we set out to investigate insight into perceptual reality monitoring errors. To this end, we asked participants to indicate confidence in a perceptual detection task while they simultaneously also imagined the stimuli they had to detect. In line with previous studies, we first observed a decrease in detection criterion during congruent imagery, indicating that participants more often reported seeing a stimulus. This is in line with imagery adding sensory evidence to both the noise and signal distributions, indicating that imagery can sometimes be mistaken for perception. We extended a hierarchical Bayesian model of confidence to characterize metacognitive insight into these perceptual reality monitoring errors. We reasoned that if participants have insight into the increase in sensory evidence due to imagery, they would alter their confidence criteria relative to their decision criteria to become less confident in “presence” responses during congruent imagery. In contrast, if participants have no insight into the effect of imagery, their confidence criteria would follow their decision criteria, leading to more confident “presence” responses. Over two experiments, we showed that confidence criteria moved in tandem with the decision criterion shift, indicating a lack of awareness of the effect of imagery on perceptual presence responses. In Experiment 2, there was also an *increase* in confidence for presence responses during congruent imagery. Interestingly, however, offline queries indicated some level of global insight into the influence of imagery on perceptual detection, but this insight was unrelated to perceptual decisions and confidence ratings. Together, our results demonstrate a lack of local insight into mistaking imagination for reality at the level of confidence ratings.

The observation that congruent imagery increases both perceptual presence responses and confidence in those responses is in line with the idea that imagery can function as perceptual evidence (Dijkstra et al., 2022; Dijkstra et al., 2021). Specifically, while an imagery-induced increase in “presence” responses can be equally explained by a stronger perceptual signal or a shift in the decision criterion, the corresponding shifts in confidence criteria break this indeterminacy in favor of a perceptual account (Gallagher et al., 2019). This idea is further supported by neuroimaging studies showing that imagery is associated with perception-like neural representations throughout the visual cortex (Albers, Kok, Toni, Dijkerman, & De Lange, 2013; Dijkstra, Bosch, & van Gerven, 2019; Pearson, 2019; Ragni, Tucciarelli, Andersson, & Lingnau, 2020). Sensory activation during imagery tends to be much weaker than during externally triggered perception, which might be why we generally do not mistake our imagination for reality (Dijkstra & Fleming, 2023; Koenig-Robert & Pearson, 2021). However, our results indicate that in ambiguous contexts, imagery-induced sensory signals might be erroneously judged as real.

Furthermore, in Experiment 2, we found that congruent imagery was associated with an increase in high-confidence false alarms. High-confidence false alarms have recently been proposed as a proxy for studying hallucinations in nonhuman animals, with studies in mice linking these percepts to elevated striatal dopamine (Schmack, Bosc, Ott, Sturgill, & Kepecs, 2021). In that context, hallucinations were induced by either manipulating perceptual expectation or reward expectation. To what extent imagery-induced hallucinations rely on similar mechanisms is unclear. One possibility is that imagery functions as a perceptual expectation, increasing the prior for imagined content. However, contrary to the usual (Bayesian) function of expectation, imagery tends to be used to generate perceptual information about stimuli that we know are not present in the environment (Kosslyn, Ganis, & Thompson, 2001). Future research is necessary to investigate how these different types of hallucinations are related.

Our findings demonstrate the multidimensional nature of insight. Despite the fact that perceptual reality judgments and confidence ratings in those judgments indicated an absence of insight, participants were nevertheless able to accurately indicate that congruent imagery would make them more likely to report perceptual presence in response to postexperiment questioning, suggesting some more global form of insight. However, these offline responses were unrelated to online perceptual reality judgments or confidence ratings, suggesting that these two forms of insight are driven by different factors. Offline insight judgments might, for example, rely more on abstract knowledge rather than direct perceptual experience during the task. This dissociation might in turn be related to a distinction between perception of reality and beliefs about reality (Dijkstra et al., 2022).

One recent study aimed to directly dissociate perceptual reality and beliefs about reality in the context of a visual illusion (Mihali, Broeker, Ragalmuto, & Horga, 2022). Prior to the experiment, participants were informed about how the illusion worked and then, in separate blocks, had to indicate their perceptual experiences (“what do you see”) and their beliefs (“what do you believe is presented on the screen”) while rating confidence in both. The results indicated that confidence ratings always tracked the first-order decision but that, in contrast to perceptual judgments, the belief judgments reflected insight into the visual illusion (Mihali et al., 2022). In our study, we asked participants “was there a grating on the screen?” which could refer either to their belief or to their perception. Future research is necessary to further disentangle these two levels of reality monitoring during simultaneous imagery and perception.

Similar dissociations between different levels of insight have been documented in relation to functional cognitive disorders—conditions in which patients believe that their cognitive functioning has declined despite intact performance on cognitive tasks (Pennington et al., 2015). A recent study of patients with functional memory disorder showed that despite a global belief of low memory performance, online confidence ratings during a memory task demonstrated intact metacognitive sensitivity (Bhome, McWilliams, Huntley, Fleming, & Howard, 2019). The authors hypothesized that this dissociation might be due to a disconnection syndrome in which global priors are unable to influence local confidence ratings. A similar mechanism might be at play in the current context, where the drivers of offline insight are unable to influence local perceptual reality judgments on individual trials.

In conclusion, by extending a hierarchical Bayesian model of metacognition to characterize confidence judgments during a simultaneous imagery and perceptual detection task, we reveal an absence of local insight into mistaking imagination for reality. However, at a global level, participants were able to indicate how imagery influenced their perceptual responses at the end of the experiment. Future research is necessary to investigate these different levels of insight and how they relate to disorders of perceptual reality monitoring.

## Supplementary Material

Supplement 1

Supplement 2
