# Supplementary figures and images for: Confidence ratings do not distinguish imagination from reality
Source: J Vis. 2024 May 30;24(5):13. doi: 10.1167/jov.24.5.13 (PMC11146086; doi:10.1167/jov.24.5.13)

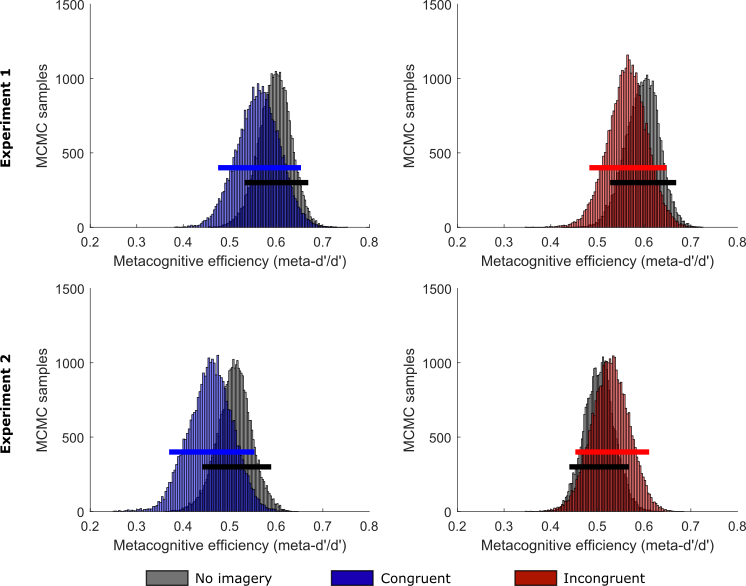

Supplement: Supplement 1 [file jovi-24-5-13_s001.png]

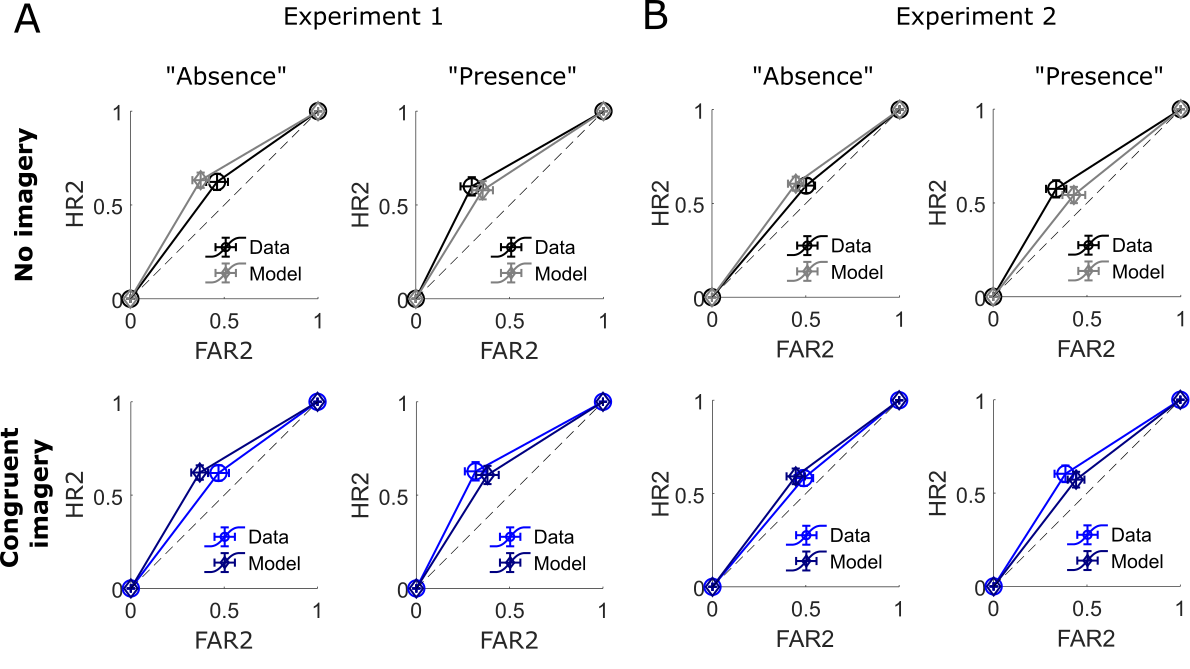

Supplement: Supplement 2 [file jovi-24-5-13_s002.png]
